# Behavioural inhibition and early neural processing of happy and angry faces interact to predict anxiety: a longitudinal ERP study

**DOI:** 10.1016/j.dcn.2023.101207

**Published:** 2023-02-02

**Authors:** Holly Rayson, Zoe J. Ryan, Helen F. Dodd

**Affiliations:** a Institut des Sciences Cognitives Marc Jeannerod, CNRS / Université Claude Bernard Lyon, France; b School of Psychology and Clinical Language Sciences, University of Reading, UK; c Children and Young People’s Mental Health Research Collaboration (ChYMe), Exeter Medical School, University of Exeter, UK

**Keywords:** Attention bias, Behavioral inhibition, EEG, ERP, Anxiety, Longitudinal

## Abstract

Limited prospective research has examined whether attention biases to emotion moderate associations between Behavioural Inhibition (BI) and anxiety in preschool-aged children. Furthermore, there has been an over-reliance on behavioral measures in previous studies. Accordingly, we assessed anxiety in a sample of preschool-aged children (3–4 years) at baseline, and again approximately 6 and 11 months later, after they started school. At baseline, children completed an assessment of BI and an EEG task where they were presented with angry, happy, and neutral faces. EEG analyses focused on ERPs (P1, P2, N2) associated with specific stages of attention allocation. Interactions between BI and emotion bias (ERP amplitude for emotional versus neutral faces) were found for N2 and P1. For N2, BI was significantly associated with higher overall anxiety when an angry bias was present. Interestingly for P1, BI was associated with higher overall anxiety when a happy bias was absent. Finally, interactions were found between linear time and happy and angry bias for P1, with a greater linear decrease in anxiety over time when biases were high. These results suggest that attention to emotional stimuli moderates the BI-anxiety relationship across early development.

## Highlights

• Developmental findings concerning attention biases, BI, and anxiety are scarce and inconsistent.
• More direct physiological measures, younger samples, and longitudinal designs can address this.
• Here, P1 responses to happy faces and N2 responses to angry faces moderated the BI- anxiety relationship.
• P1 responses to both happy and angry faces predicted a linear decrease in anxiety over time, irrespective of BI.
• Attention processes across early childhood moderate the BI-anxiety relationship.

Following cognitive models of anxiety that posit attention processes as critical in the development and maintenance of anxiety (Beck and Clark, 1997, Mathews and Mackintosh, 1998, Williams et al., 1988), a substantial body of research has examined whether anxiety is associated with preferential attention to threat. In adults and children, there is evidence supporting this association (Dudeney et al., 2015), although not consistently (Kruijt et al., 2019), and some research finds anxiety is linked to attention *away* from threat (Shechner et al., 2012). Alongside research examining direct associations between attention bias and anxiety, there is emerging evidence that attention bias to threat might moderate the association between Behavioural Inhibition (BI) and anxiety (Nozadi et al., 2016, Pérez-Edgar et al., 2011, Pérez-Edgar et al., 2010, White et al., 2017). BI is a temperament style characterised by withdrawal and wariness in unfamiliar, novel situations (Kagan et al., 1984) that has consistently been linked to risk for subsequent social reticence and anxiety (Chronis-Tuscano et al., 2009, Clauss and Blackford, 2012, Hudson et al., 2019). However, not all inhibited children go on to experience anxiety (Fox et al., 2001), and attention bias to threat may act as a moderator of the BI-anxiety relationship (Nozadi et al., 2016, Pérez-Edgar et al., 2011, Pérez-Edgar et al., 2010, White et al., 2017). There are some limitations to this work though, including a general reliance on behavioral measures, limited prospective research, and little research focusing on preschool-aged children.

Anxiety-linked attention biases have been hypothesised in relation to the initial orienting stage of attention (vigilance model, Beck and Clark, 1997), the later executive control stage of attention (delayed disengagement model, Fox et al., 2001) and both early and late processes (vigilance-avoidance model, Mogg & Bradley, 1998). Behavioural measures of attention bias typically rely on reaction times, which provide an indirect measure of attentional processes, and there are concerns about reliability (Brown et al., 2014, Rodebaugh et al., 2016). Furthermore, such tasks provide only a limited insight into these earlier versus later attention processes because they typically assess attention at a specific timepoint following stimulus onset, and cannot therefore capture the dynamic nature of attention processes as they unfold over time.

Some of the issues outlined above can be addressed by using more direct measures of attention-related processes such as eyetracking (Clauss et al., 2022) and electroencephalography (EEG) (Gupta et al., 2019, Kappenman et al., 2014). Studies using eyetracking to examine a link between anxiety and attention bias to threat in youth samples have provided mixed findings (Shechner et al., 2013, Gamble and Rapee, 2009, Schmidtendorf et al., 2018, Stuijfzand et al., 2020, Lisk et al., 2020). Notably, one eyetracking study found that attention bias to threat was associated with higher anxiety over the transition to school, only for BI children (Dodd et al., 2020). EEG, a non-invasive technique suitable for measuring neural activity in young children, can be used to measure attention-related event related potentials (ERPs). ERPs can be linked to specific stages of attention allocation and are considered one of the most reliable measures of attention processes associated with threat-related stimuli biases (Reutter et al., 2017, Torrence and Troup, 2018, Wieser and Keil, 2020). Although some progress has been made using EEG to examine anxiety-linked attention processes in adults (Bar-Haim et al., 2005) and older children (Thai et al., 2016), very few studies have utilized ERPs to investigate anxiety-linked attention bias in preschool-aged children.

A number of ERP components associated with attentional processes are particularly relevant to research on the aetiology of paediatric anxiety. Amplitude of the posterior P1 component, which is generated in the primary visual cortex when a visual stimulus is detected (Luck, 2014), is greater in response to emotional stimuli (Itier and Taylor, 2004), and has been linked to anxiety in adults when measured in response to threat-related facial expressions (e.g (Luck, 2014, Itier and Taylor, 2004). P1 findings in children are more inconsistent. Greater P1 amplitude to angry and fearful versus neutral faces has been associated with anxiety in youth aged 6–15 years (Bechor et al., 2019, Willner et al., 2020), and others have found a more general association between P1 responses to facial expressions and anxiety at 8–12 years (Hum et al., 2013a, Hum et al., 2013b). Such findings suggest that greater automatic attentional capture of threat-related stimuli could play a role in early-emerging anxiety disorders, but other studies in children aged 6–12 found no relation between P1 responses to faces and childhood anxiety (e.g (Chronaki et al., 2018), as well as no moderating effects of BI (Thai et al., 2016).

The generation of the mid-stage anterior P2 ERP component likely involves frontal regions such as the anterior cingulate cortex, with P2 amplitude again greater in response to emotional stimuli (Potts and Tucker, 2001, Carretié et al., 2004). P2 responses are thought to indicate attentional resource capture (Schupp et al., 2004), and though less examined than other components, adult studies of anxiety have noted a relationship with P2 responses to different facial expressions (e.g (Falkenstein et al., 1999, Van Veen and Carter, 2002, Yeung and Cohen, 2006). Thai et al (Thai et al., 2016). found that in children aged 9–12 years, P2 amplitude to faces moderated the relationship between childhood BI and anxiety, with a greater P2 amplitude related to less concurrent anxiety symptoms. This suggests that greater allocation of attentional resources during evaluation of faces may serve as a compensatory mechanism in very inhibited children, but replication of these results is warranted.

The later-stage anterior N2 component is generated in the anterior cingulate and orbitofrontal cortex (Bekker et al., 2005), and is linked to more effortful and top-down processes such as attentional control (Falkenstein et al., 1999, Van Veen and Carter, 2002, Yeung and Cohen, 2006). N2 responses may reflect effort diverting attention away from threat in anxious adults (Dennis and Chen, 2007, Eldar and Bar-Haim, 2010). In children aged 8–12 years, for example, BI is associated with social reticence only when N2 amplitudes to non-emotional stimuli are greater, with N2 amplitude to calm faces also associated with greater anxiety (Hum et al., 2013a). In contrast, Thai et al (Thai et al., 2016). found no direct association between N2 amplitude in response to faces and anxiety, but did find that a greater N2 amplitude was linked to greater behavioural threat avoidance in those higher in BI.

Although the majority of research has focused on threat biases, some initial findings indicate that attention bias to positive stimuli might be protective against anxiety. One study showed that training anxious 7–13-year-olds to attend to happy faces led to a decrease in anxiety (Waters et al., 2013), while BI children with an attention bias for happy faces have been shown to have lower levels of anxiety at 7 years (White et al., 2017). This finding was recently replicated for preschool children, with BI interacting with an attention bias towards happy faces to predict future anxiety (Dodd et al., 2020). A bias towards happy faces also moderates risk for subsequent internalizing problems in children aged 8–12 years who experienced early institutionalization (Troller-Renfree et al., 2017, Troller‐Renfree et al., 2015). A few adult studies have linked ERP responses to happy faces with anxiety (Rossignol et al., 2013, Morel et al., 2014), but use of ERPs in longitudinal studies of temperament and anxiety are now needed to clarify the role of positive biases in the early emergence of anxiety in childhood.

To summarise, various ERP components provide insight into attentional processes of relevance to anxiety-linked attention biases, but research with child samples remains rare, and prospective research with preschool-aged samples is particularly scarce. This is important because developmental pathways to anxiety disorder often begin in the preschool years, with some children receiving a diagnosis of clinical anxiety as young as age 3 (Luby, 2013). The preschool period is also a central focus for anxiety prevention programmes (e.g. Turtle/Cool Little Kids) (Chronis‐Tuscano et al., 2022). The aim of the present research was therefore to evaluate whether attention-linked ERP responses to angry and happy faces moderate the longitudinal association between BI and anxiety in preschool-aged children (approximately 4-years-old). We assessed ERPs, BI and anxiety at baseline, and then conducted two follow-up assessments of anxiety symptoms. The follow-ups were conducted during the first half of each child’s first and second term of school respectively. These timepoints were selected to capture response to and recovery from a universal stressor (starting school; see (Leblond et al., 2022)).

We hypothesized that an early attention bias to angry faces, characterised by greater P1 and N2 amplitude to angry faces relative to neutral, would interact with BI to predict anxiety, and with BI and time to predict trends in anxiety over the three timepoints. Specifically, we expected that greater attention to angry faces would be associated with higher anxiety and a greater increase in anxiety over time, and that this effect would be stronger in higher BI than lower BI children. For attention bias to happy faces, we hypothesized the opposite patterns of BI-anxiety moderation over time, such that bias for happy faces would be associated with less anxiety, especially in higher BI children. As very little research has focused on P2, we tentatively hypothesized that the response of this ERP component to angry faces would moderate the BI-anxiety relationship over time, but we did not predict a specific direction of effect.

## 1 Methods

### 1.1 Participants

A sample of 180 typically developing preschool-aged children (3.42–4.83 years (M = 3.97, SD = 0.25; 90 female)) were recruited via preschools, public advertising, social media and word of mouth to take part in a project about children’s emotions when they start school. Most children were described by their parent as being White British (83.3%). See Supplementary Information (SI) for further sample information. Follow-ups took place during the first and second terms of school. The average time between baseline and follow-ups was 6.49 months for the first follow-up (SD = 2.45 months; range 3–12 months) and 10.77 months for the second follow-up (SD = 2.41 months; range 7–16 months). All participants were invited to complete the EEG task and 97 completed the task (71 had useable data for analysis after pre-processing, as outlined below). Reasons for not completing the EEG included can be found in the supplementary information (SI; Table S2).

### 1.2 Procedure baseline

Procedures were approved by the University of Reading Research Ethics committee (UREC 16/56). Parents were provided with detailed information about the project and arranged to visit the University with their child for a lab session which lasted around 3 h in total. At the beginning of the lab session parents provided written consent. Participants were shown a video explaining the procedures and we sought their assent using a traffic light system (red = no, yellow = question or unsure, green = happy to take part). During the lab session families completed the tasks reported here, as well as other tasks as part of the wider study. A maximum of 5 blocks of the EEG task were completed but if participants requested to stop sooner the task was terminated. One of the researchers sat with the participant throughout the task. During this lab session, BI was also assessed using the scores the Behavioral Inhibition Questionnaire (BIQ) and LabTab, with anxiety assessed using The Preschool Anxiety Scale (PAS). At the end of the session participants were thanked for their participation and given a small gift; parents were given £ 35.

### 1.3 Procedure follow-ups

Parents consented to take part in two follow-up stages during the baseline session. Both follow-up stages required parents to complete a set of online questionnaires. Parents were emailed approximately one week after their child started school asking them to complete the PAS questionnaire. A reminder email was sent two weeks later, and the online questionnaire was closed approximately 6 weeks after the children’s first day at school. For the second follow-up, parents were emailed approximately one week after their child started their second term of school and, similarly, the PAS questionnaire was closed approximately 6 weeks later.

### 1.4 Apparatus and materials

***Overall BI score.*** Children were given an overall BI score by combining their total score on the parent-report Behavioral Inhibition Questionnaire (BIQ) (Bishop et al., 2003) and their total observed BI score from the LabTab (Gagne et al., 2011) (see SI).

***Parent report of anxiety symptoms.*** The Preschool Anxiety Scale (PAS) (Spence et al., 2001) was used to assess symptoms of child trait anxiety (see SI).

### 1.5 EEG Task

Participants completed a passive viewing task presented using E-prime version 2.0.10.356 (Psychological Software Tools, Pittsburgh, PA, USA). Stimuli consisted of three male and three female individuals each displaying happy, angry, and neutral expressions retrieved from Radboud Faces Database (Langner et al., 2010). The faces were converted to greyscale, matched for mean luminance and RMS contrast, and their pupil position was aligned. They were windowed using an elliptical mask with a Gaussian blur and presented on a mid-grey background. The images were 224 pixels horizontally by 323 pixels vertically. They were presented centrally on a sonic monitor (VA2413wm), which measured 21.5 in. horizontally by 11.5 in. vertically, with 1920 × 1080 resolution. The programmed task consisted of five blocks with 36 trials in each but fewer blocks were completed if the participant did not provide assent to continue after each block. A blank screen was jittered for 1000–1200 ms, followed by a fixation cross jittered for 800–1000 ms to enable baseline sampling of 100 ms before stimuli onset. One emotional face (happy, angry, or neutral) was presented per trial for 2500 ms. Images were presented so that no two consecutive images were identical, but randomised otherwise.

### 1.6 EEG recording and pre-processing

EEG was recorded using a 128-channel HydroCel Geodesic Sensor Net (Electrical Geodesics, Inc., Eugene, OR), with the vertex (Cz) electrode used for online reference. Data were sampled at 250 Hz using EGI’s Net Station (v5) software, and impedances were kept below 50 kΩ. After recording, the data were exported using NetStation software for offline processing. Data were then pre-processed using the MADE pipeline (https://github.com/ChildDevLab/MADE-EEG-preprocessing-pipeline; (Debnath et al., 2020)) and MatLab 2018a. More details can be found in the SI. Participants with < 10 remaining trials per condition after pre-processing were excluded, leaving 71 participants in total. Trials remaining per participant: angry, M = 24.64, SD = 10.15; happy, M = 24.47, SD = 9.99; neutral, M = 24.49, SD = 10.91.

**Fig. 1:**
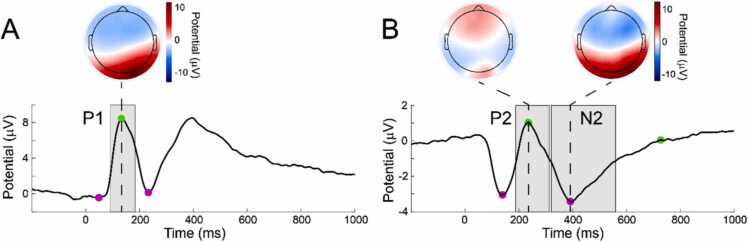
Grand average waveforms and scalp topographies for the occipital P1 component *(A)* and for the fronto-central P2 and N2 components *(B)*, time-locked to stimulus presentation.

Based on similar research (e.g (Potts and Tucker, 2001). and inspection of the grand average waveforms, the P1 ERP component was quantified as the average mean amplitude over an occipital electrode cluster (R Core Team, 2022, Bates et al., 2014, Long, 2019, Gunther et al., 2022, Dodd et al., 2015, Henderson, 2010, Fox et al., 2021, Troller-Renfree et al., 2019; 82,83,84,89,90), and the P2 and N2 ERP components as the average mean amplitudes over a fronto-central electrode cluster (3, 4, 5, 10, 11, 12, 16, 18, 19, 20, 23, 24, 27, 28, 117, 118, 123, 124). Time windows for analysis of each ERP component were determined empirically using the aggregate grand average from trials (AGAT), allowing data driven time window selection without inflating Type I errors (Brooks et al., 2017); see SI for more information. Finally, for each component, outlier participants with greater than 3 times the median absolute deviation from the median were removed within condition (P1 angry = 4, happy = 4, neutral = 2; P2 angry = 2, happy = 2, neutral = 6; N2 angry = 1, happy = 1, neutral = 6) (Leys et al., 2013). See Fig. 1 for grand average waveforms and scalp topographies for each ERP component.

### 1.7 Statistical analysis

To investigate condition differences in mean amplitude of each ERP component (P1, P2, N2), separate linear mixed models were run with condition (angry/happy/neutral) as a fixed effect and subject-specific offsets as a random effect. All participants (N = 71) were included in these analyses. *P*-values for fixed effects were obtained using Type II Wald *F* tests, and significant main effects of condition were followed up by planned pairwise comparisons of least square means. Pairwise comparisons were Tukey-corrected for multiple comparisons, and degrees of freedom were approximated using the Kenward-Rogers method. For ease of interpretation, we reversed the polarity of N2 (i.e. from negative to positive, with more positive values thus representing a greater (more negative) N2 amplitude).

To examine whether the degree of bias towards angry and happy faces in ERP responses related to anxiety scores at each assessment time-point, angry-bias and happy-bias scores were calculated separately for each ERP component (P1, P2, N2) and participant using the following formulae to obtain normalized differences between component mean amplitudes in the emotion and neutral conditions (Xie et al., 2021): i) Angry-bias score = (Angry – Neutral) / (|Angry| + |Neutral|); Happy-bias score = (Happy – Neutral) / (|Happy| + |Neutral|). Therefore, for all ERP components, a positive score represents a bias towards angry or happy versus neutral. Hierarchical growth curve analyses were run for each ERP component (P1/P2/N2) to investigate how ERP biases may moderate the BI-anxiety relationship over time, with separate models run for happy and angry biases. Emotion ERP component bias, BI (centred), and their interaction were included as fixed effects, as were linear and quadratic orthogonal polynomial time terms (poly1 and poly2, respectively), and their interactions with ERP bias and BI. Subject-specific offsets were included as a random effect. *P*-values for each factor and their interactions were obtained using Type III Wald *F* tests. We probed significant interactions between continuous variables and polynomials using the Johnson-Neyman technique (Johnson and Neyman, 1936); see SI for details. Those who had missing BI data (N = 1), and/or had missing anxiety data (baseline, N = 1; follow-up 1, N = 6; follow-up 2, N = 4) were excluded from these analyses (see Results section for specific numbers of participants included in each growth curve model). Note, post-hoc power analyses were performed for all significant main effects and interactions, with results presented in the SI.

Analyses presented here were performed using R (v4.1.1; (R Core Team, 2022)) and the lme4 (v1.1.27; (Bates et al., 2014)), emmeans (v1.5.3; (Lenth, 2020)), MASS (v7.3–53; (Venables, 2002)), and interactions (v1.1.0; (Long, 2019)) packages.

## 2 Results

Descriptive statistics for each variable and the correlations between variables are presented in Table 1. No significant correlations were found between any of the ERP bias scores and BI or anxiety at any timepoint (*p* > .05). Anxiety symptoms were highly correlated at each time point, and with BI at baseline and follow-up 1.

**Table 1:** Means, standard deviations, ranges and bivariate correlations for the sample of participants included in the analyses presented below. The N for each correlational analysis is given in ().

	*M (SD)*	1	2	3	4	5	6	7	8	9	10
1. P1 angry bias	0.06 [0.37] -1–1	1.00 (65)									
2. P1 happy bias	0.10 [0.39] -1–1	0.22 (61)	1.00 (63)								
3. P2 angry bias	0.18 [0.68] -1–1	-0.10 (59)	0.15 (57)	1.00 (61)							
4. P2 happy bias	0.16 [0.75] -1–1	0.08 (59)	-0.49 ***(57)	0.18 (60)	1.00 (62)						
5. N2 angry bias	0.10 [0.64] -1–1	0.22 (60)	0.00 (57)	-0.32 * (58)	-0.03 (58)	1.00 (62)					
6. N2 happy bias	0.04 [0.64] -1–1	-0.12 (59)	0.28 * (56)	-0.18 (57)	-0.42 *** (59)	-0.36 ** (61)	1.00 (62)				
7. BI	0.07 [0.77] -1,52–2.62	0.11 (65)	0.14 (63)	0.08 (61)	-0.02 (62)	-0.02 (62)	0.06 (62)	1.00 (69)			
8. Baseli-ne PAS	22.96 [13.81] 1–78	0.05 (65)	0.18 (63)	-0.01 (61)	-0.11 (62)	-0.03 (62)	0.01 (62)	0.48 *** (69)	1.00 (69)		
9. Follow -up 1 PAS	20.88 [13.24] 0–58	-0.05 (60)	-0.11 (58)	-0.06 (57)	0.04 (58)	-0.23 (58)	-0.02 (58)	0.39 ** (64)	0.69 *** (64)	1.00 (64)	
10. Follow -up 2 PAS	19.86 [14.32] 0–91	-0.07 (62)	-0.14 (60)	-0.05 (58)	0.05 (59)	-0.03 (59)	0.15 (59)	0.18 (66)	0.56 *** (66)	0.78 *** (66)	1.00 (66)

*Note.* PAS = Preschool Anxiety Scale; BI = Behavioral Inhibition.

*indicates p < .05. * indicates p < .01. * * indicates p < .001 * ** .

### 2.1 ERP components: condition differences

**Fig. 2:**
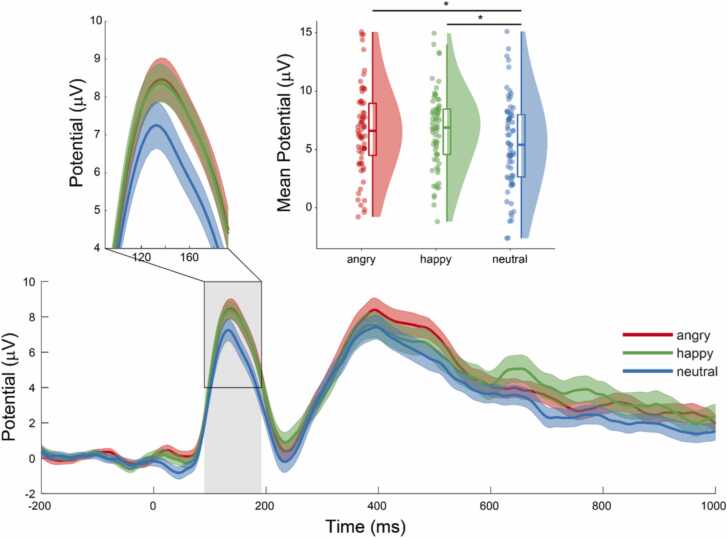
Grand average ERP waveform from the occipital electrode cluster in response to the different conditions. The peak amplitude of the grand average P1 component in the angry and happy face conditions was greater than in the neutral face condition *(top, left)*. Mean P1 amplitude in the angry and happy conditions was significantly greater than in neutral *(top, right)*. Dots represent the mean amplitude for each participant. Box plots show the median and the first and third quartiles, and the shaded area shows the distribution density.

For the P1 model, a significant main effect of condition was revealed [*F*[2] = 4.52, *p* = 0.01], with mean amplitude of P1 in angry [*t*(131) = 2.5, *p* = 0.04] and happy [*t*(132) = 2.68, *p* = 0.02] conditions significantly greater than in neutral (see Fig. 2). No differences between conditions were found for P2 or N2 components.

### 2.2 Predicting anxiety symptoms

Across all models, BI was a significant predictor of anxiety symptoms, with higher BI linked to greater anxiety scores (all *p* < 0.005). Note, no significant effects involving ERP biases were found for the P2 happy bias (n = 62) or P2 angry bias models (n = 61), or effects involving the quadratic time polynomial (poly2) in any model. Additionally, no three-way interactions were found between BI, ERP components, and either time polynomial.

#### 2.2.1 P1 models

**Fig. 3:**
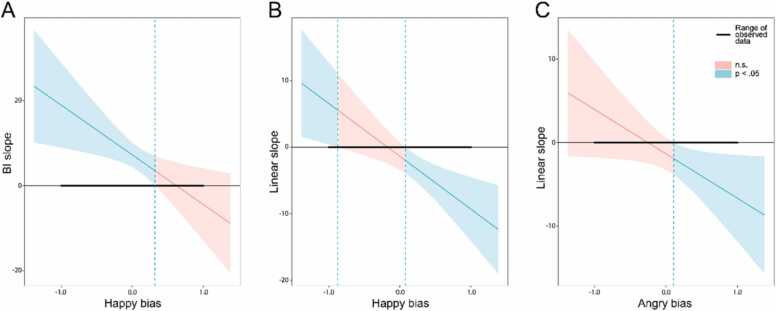
Johnson–Neyman plots illustrating results probing significant interaction effects P1 models. The plots show the slope of the relationship between BI or poly1 and anxiety, varying with levels P1 emotion bias. The bold horizontal lines show the range of the observed data, and the shaded areas indicate at which bias levels the relationship between BI/poly1 and anxiety was significant (*p* < 0.05; shaded blue regions) and non-significant (n.s.; shaded red regions). P1 happy-bias moderated the BI-anxiety relationship *(A)*, the slope of this relationship significantly differed from zero when P1 happy bias was ≤ 0.32; BI was associated with more anxiety most strongly at the most negative levels of happy bias. P1 happy bias moderated a decrease in anxiety over time *(B)*. The linear decrease in anxiety over time significantly differed from zero when P1 happy bias was ≥ 0.08; at higher levels of bias, there was a stronger linear decrease in anxiety over time. Furthermore, the opposite pattern was seen at negative levels of happy bias; where P1 happy bias was ≤ −0.88, there was a linear increase in anxiety between baseline and follow-up 2. P1 angry bias also moderated a decrease in anxiety over time *(C)*. The linear decrease in anxiety over time was significant where P1 angry bias was ≥ 0.11; at higher levels of bias, there was a stronger linear decrease in anxiety over time.

For the P1 happy bias model (n = 63), there were also significant interactions between BI and happy bias [*F*[1] = 6.86, *p* = 0.01] and happy bias and poly1 [*F*[1] = 9.33, *p* = 0.003]. As shown in Fig. 3A, probing this interaction between BI and happy bias with the Johnson-Neyman procedure revealed that at lower (and negative/absent) levels of happy bias, there was a positive relationship between BI and anxiety (i.e. where happy bias was ≤ 0.32, higher BI was related to more anxiety). Probing the interaction between happy bias and poly1 (Fig. 3B) revealed that a greater happy bias was linked to a decrease in anxiety over time (i.e. where happy bias was ≥ 0.08, there was a linear decrease in anxiety between baseline and follow-up 2). Additionally, a negative happy bias was linked to an increase in anxiety over time (i.e. where happy bias was ≤ −0.88, there was a linear increase in anxiety between baseline and follow-up 2). For the P1 angry bias model (n = 65), there was a significant interaction between angry bias and poly1 [*F*[1] = 4.66, *p* = 0.03]. As shown in Fig. 3C, probing this interaction revealed that a higher level of angry bias was linked to a decrease in anxiety over time (i.e. where angry bias was ≥ 0.11, there was a linear decrease in anxiety between baseline and follow-up 2).

#### 2.2.2 N2 models

**Fig. 4:**
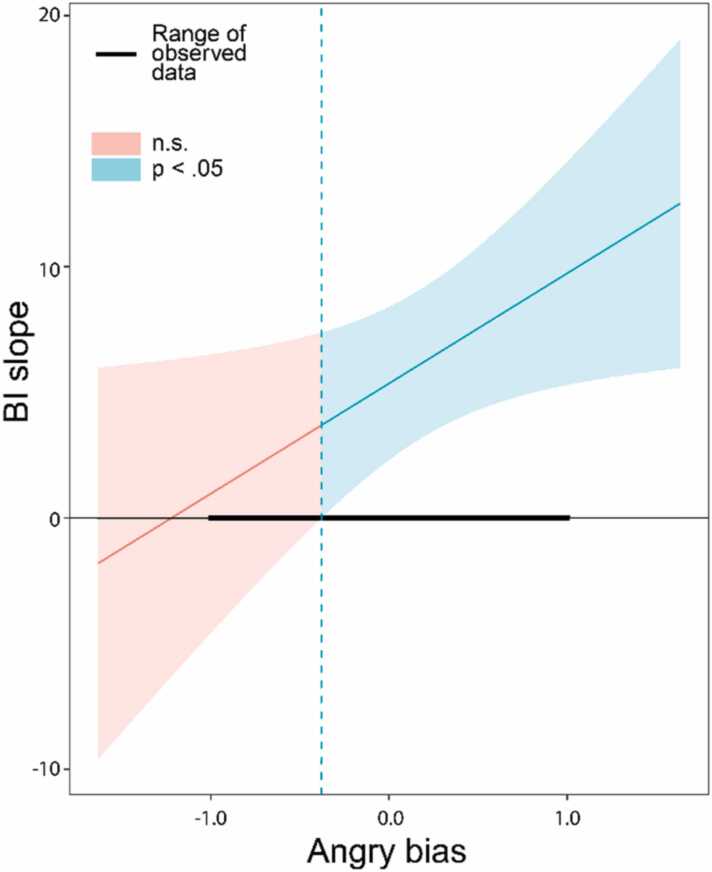
Johnson–Neyman plot illustrating results probing significant interaction effects for the N2 angry bias model. The plot shows the slope of the relationship between BI and anxiety, varying with levels N2 emotion bias. The bold horizontal lines show the range of the observed data, and the shaded areas indicate at which bias levels the relationship between BI and anxiety was significant (*p* < 0.05; shaded blue regions) and non-significant (n.s.; shaded red regions). N2 angry bias moderated the BI-anxiety relationship. BI was linked to more anxiety when angry bias was ≥ −0.38; BI was associated with more anxiety most strongly at the most positive levels of N2 angry bias.

For the N2 happy bias model (n = 62), no significant effects of bias were revealed. For the N2 angry bias model (n = 62), an interaction between BI and angry bias was revealed [*F*[1] = 4.67, *p* = 0.03]. As shown in Fig. 4, probing this interaction revealed that a higher levels and angry bias, there was a positive relationship between BI and anxiety (i.e. where angry bias was ≥ −0.38, higher BI was linked to more anxiety). No effects of poly1 were revealed.

## 3 Discussion

This research aimed to examine whether ERP responses to emotional faces moderated the longitudinal association between BI and anxiety in preschool-aged children. We first hypothesized that an early attention bias to angry faces, characterised by greater P1 and N2 amplitude to angry faces relative to neutral, would interact with BI to predict anxiety and also interact with BI and time to predict trends in anxiety over the three timepoints. The results only provided partial support for these hypotheses. For N2, an interaction between angry bias and BI was found; as baseline N2 angry bias increased there was a stronger, positive association between BI and anxiety that remained stable over time. No angry bias by BI interaction was found for P1, and no three-way interactions between bias, BI and time were found for either P1 or N2. Our second hypothesis was that an early attention bias to happy faces would interact with BI to predict anxiety and interact with BI and time to predict trends in anxiety over the three timepoints. Again, partial support for these hypotheses was found. P1 happy bias interacted with BI to predict anxiety; as P1 happy bias at baseline decreased there was a stronger, positive association between BI and anxiety that remained stable over time. No interaction was found for N2 happy bias and no three-way interactions were found. In relation to P2, we tentatively hypothesised that angry and happy bias for P2 would interact with BI to predict anxiety, but no main effects nor interactions involving P2 were found. Overall, our findings support previous research indicating that attention processes in response to emotional stimuli moderate the BI-anxiety relationship (Nozadi et al., 2016, Pérez-Edgar et al., 2011, Pérez-Edgar et al., 2010, White et al., 2017, Gunther et al., 2022), and extend initial findings showing that attention bias to positive stimuli may be adaptive for young children (Dodd et al., 2020).

The three study timepoints were originally designed to capture anxiety symptoms before, during and after a naturalistic stressor (starting school). However, the descriptive statistics and effects of time included in our analyses suggest that the expected pattern of elevated anxiety during the transition to school, followed by recovery, was not found. Instead the main effects of linear and quadratic time were not significant, indicating relatively stable anxiety across the three timepoints. The descriptive statistics indicate that, if anything, anxiety slightly decreased over the three timepoints. The results need to be interpreted with this general stability in anxiety symptoms in mind.

We did not find the hypothesised three-way interactions; attention bias moderated the association between BI and anxiety but did not moderate the association between BI and anxiety *trajectories.* We did however find interactions between both P1 angry bias and P1 happy bias and the linear time term, with both results indicating that having a stronger bias to emotional faces is associated with a stronger linear decrease in anxiety over time. Although these effects were not hypothesised, this suggests that, for preschool-aged children, having stronger early, automatic visual processing of emotional faces is linked to decreasing anxiety trajectories, irrespective of those children’s BI levels. Previous research has shown that bias to angry and happy faces is normative in preschool children (Stuijfzand et al., 2020, Dodd et al., 2015), which seems to be in keeping with these findings; children who do not show these normative biases do not show an adaptive decrease in anxiety over time. This is an important finding because it demonstrates that attention bias to threat may not always be problematic and, in fact, may be important for young children’s healthy development. It also highlights that further exploration of a potential protective influence of bias for positive stimuli is warranted.

Returning to the interactions between bias and BI, the happy-bias interaction with BI was only found for P1, which is indicative of early, automatic visual processing. The findings are therefore not indicative of a frontally-mediated emotion regulation strategy. The interaction between BI and happy bias is similar to findings from research using the dot-probe task (White et al., 2017), as well as our previous eyetracking work (Dodd et al., 2020). Consistent with our findings, in both previous studies, bias to happy faces was protective for high BI children. It is noteworthy that it was the *absence* of a P1 happy bias that predicted a positive relationship between BI and anxiety in the current study. This is in keeping with the above point that having a stronger response to happy faces, relative to neutral, in early, automatic visual processing may be adaptive; when BI children do not exhibit this bias they are at risk for anxiety, when they do they are not at risk for anxiety. An important caveat in relation to consistency with previous research is that the current participants took part in our previous eyetracking study (Lisk et al., 2020), so the result requires replication in an independent sample. Importantly though, the eyetracking task, which was separate from the EEG task, used faces from a different database and the task was very different, with gaze recorded whilst participants viewed pairs of emotional-neutral faces for 1500 ms. The timing on which the eyetracking analyses were based was thus distinct from the P1, which peaked around 140 ms. Attention bias to positive stimuli is not frequently examined in child anxiety research, but our findings are consistent with other existing research in this area (e.g (Troller-Renfree et al., 2017, Troller‐Renfree et al., 2015, Morel et al., 2014, Waters et al., 2008).).

We also found that N2 bias for angry faces interacted with BI to predict anxiety. N2 is an ERP component associated with cognitive control. Specifically, we found that a N2 angry bias moderated the BI-anxiety relationship across time, with a positive relationship between BI and anxiety found when angry bias was positive; i.e. the relationship between BI and anxiety was stable when N2 bias was high. This finding is consistent with previous research showing that early BI was associated with later social reticence only when children also had higher N2 amplitude on a go/no-go task (Lamm et al., 2014), and that shyness was associated with social anxiety only when children had a relatively large N2 responses on a flanker task (Henderson, 2010). Interestingly, both these studies assessed N2 on non-emotional cognitive tasks in older children, but our findings with preschool-aged children completing a passive viewing task closely replicate them. Importantly however, we only found this effect for N2 angry-bias not happy-bias, which suggests that the attention control processes engaged specifically in the presence of threat are relevant to anxiety risk. Previous research has interpreted N2 to be associated with effort to control, inhibit or divert attention away from a stimulus, with greater amplitude indicative of more effort. Our findings could therefore suggest that BI children who exert more effort in diverting attention away or inhibiting a prepotent response, whether or not they are successful, when viewing angry faces are at increased risk for anxiety in the longer-term. Therefore, for high BI children, anxiety risk appears to be lowest when children require relatively little effort to implement planful control processes. This interpretation is necessarily speculative, but the results clearly indicate that, even in young children, attentional control processes are important moderators of anxiety risk in the context of high BI, which is nicely consistent with the latest theory and research with older children (Fox et al., 2021, Troller-Renfree et al., 2019, Valadez et al., 2021, White et al., 2011).

We did not find any effects of P2. Relatively little attention bias research has examined P2 but this findings is not in accordance with the results of Thai and colleagues (Thai et al., 2016), who found P2 amplitude (to angry and neutral faces combined on a dot-probe task) interacted with BI to predict concurrent anxiety in children aged 9–12; the higher the P2 amplitude, the weaker the association between BI and anxiety. Given that P2 amplitude appears to relate to attentional resource allocation, Thai and colleagues interpreted their findings as indicating that greater allocation of resources may be protective for inhibited children, potentially counteracting earlier reactive responses, although note, they did not find any association between P1 amplitude to faces and anxiety. There are multiple potential reasons for this discrepancy in results, including the difference in participant age, examination of combined responses to angry and neutral faces rather than angry versus neutral, and the use of different tasks requiring different responses. In any case, more longitudinal research is now required to elucidate the potential moderating role of P2 in the BI-anxiety relationship across development.

Finally, although P1 angry and happy biases interacted with linear time to predict anxiety (as discussed above), no direct bivariate correlations between ERP amplitudes and anxiety or BI were found. An effect of emotion was found for P1 amplitude, with amplitude to happy and angry faces higher than amplitude to neutral faces, replicating recent research in young children (e.g (Valadez et al., 2021). No effect of emotion was found for P2 or N2, which again is relatively consistent with previous findings, although research in this area with preschool-aged children is scarce (Bar-Haim et al., 2005, Kanske et al., 2011). Therefore, our findings also emphasize the importance of looking at individual differences in developmental studies examining neural predictors of anxiety, with such differences also predicting behavioral attention biases to facial expressions in other recent research that failed to find group-level differences in ERP responses between emotions (Xie et al., 2021).

This work has a number of strengths, including the focus on preschool-aged children, which is important because pathways to anxiety begin early in life, yet attention bias research with young children remains rare. The use of ERPs rather than reliance on a behavioral task is also a strength because it allows insight into specific subcomponents of attention as well as the timeline of early attention to visual stimuli, as is the longitudinal design. The sample size is relatively large for EEG research with preschoolers compared to similar studies (see 79), but we acknowledge that we had relatively low rates of participation in our EEG task, with children and/or parents not providing assent despite us setting up the lab and study in a child-friendly way. There are a number of possible reasons for this. The most likely is that the EEG task was completed as part of a larger assessment battery during a visit to the laboratory. This meant that families had not specifically volunteered to take part in an EEG study, and relatively little time was available for second attempts. We also worked hard to ensure that our assent procedure was clear for young children, using a traffic light system for children to let us know how they were feeling about doing each stage of the research. We designed this to ensure children could tell us if they wanted to stop or did not want to do any of the tasks. As a result, a relatively high number of children may have felt able to withhold or withdraw their assent. We respected this choice and did not coerce participants to continue. Importantly, no differences were found between those whose data were included and those who did not complete the task, or whose data did not include anxiety or BI measures; however, it is also important to note that some effect sizes acquired via post-hoc power analyses for the different findings were relatively low, therefore these results now need to be replicated with larger samples (see SI for regarding analyses regarding this lack of differences between groups and power analyses). We selected P1, P2, and N2 ERP components as these appear particularly relevant for understanding early emerging anxiety based on previous literature, but a number of other ERP components have been linked to affect-biased attention and anxiety. An additional limitation is that passive viewing tasks can be difficult to interpret as it can be unclear what participants are doing whilst looking at the stimuli. This contrasts with behavioural tasks where a specific response or action is required. The task also lacks ecological validity. This is necessarily the case for the majority of EEG research, which requires carefully controlled designs, but it is important for future research to examine whether and how lab-based attention bias metrics have relevance in real-world settings.

To conclude, our results contribute to a growing body of literature showing that BI is more closely linked to anxiety in the context of other risk markers. This is the first study to demonstrate that ERPs to emotional faces moderate the BI-anxiety link in children over time, and further extends the literature by focusing on attention biases and its links to anxiety longitudinally in very early childhood. Priorities for future research include extending follow-ups into middle childhood and adolescence, when anxiety disorder onset is most common, and further measurement of ERPs over development to provide insight into how neural attention mechanisms and BI interact across time to affect anxiety risk.

## Funding

The research was funded by an ESRC Future Research Leaders grant awarded to HD (ES/L010119/1). For the purpose of open access, the author has applied a ‘Creative Commons Attribution (CC BY) licence to any Author Accepted Manuscript version arising

## Declaration of Competing Interest

The authors declare that they have no known competing financial interests or personal relationships that could have appeared to influence the work reported in this paper.

## Data Availability

The research data and analysis code supporting this publication are openly available from ReShare at: https://dx.doi.org/10.5255/UKDA-SN-853813.
