# Supplementary material for: Behavioural inhibition and early neural processing of happy and angry faces interact to predict anxiety: a longitudinal ERP study
Source: Dev Cogn Neurosci. 2023 Feb 2;60:101207. doi: 10.1016/j.dcn.2023.101207 (PMC9929676; doi:10.1016/j.dcn.2023.101207)
Supplement: Supplementary file 1 — Supplementary material [file mmc1.docx]

**Supplementary Information**

**METHODS**

**Participants**

The majority of the children were described by their parent as being White British (83.3%). The rest of the sample were described as being White but of other descent (N = 9); Indian (N = 2); Pakistani (N = 2); Chinese (N = 1); Black British (N = 2); Mixed Race (N = 8); other (N = 6). At baseline, parents were asked whether their child had any diagnosed learning difficulty, mental health problem or atypical development. Three parents answered yes: one child had an Autism Spectrum Disorder diagnosis; one had a midline cleft palate and the third had a developmental delay. The data for only one of these three children was included in the analysis; the child with the developmental delay.

Further details of demographic characteristics for both the full sample and the sample included in the analysis can be found in Table S1 below

**Table S1.** Demographic characteristics of full sample and sample included in analysis

| **Characteristic** | **Full Sample *N* (%)** | **Sample Included in Analysis *N* (%)** |
| --- | --- | --- |
| Child gender  Male  Female | 180  90 (50%)  90 (50%) | 71  28 (39%)  43 (61%) |
| Child age  3  4 | 180  119 (66%)  61 (34%) | 71  42 (59%)  29 (41%) |
| Child ethnicity  White British  White Irish  White European  White Other  Asian or Asian British (Indian origin)  Asian or Asian British (Pakistani origin)  Asian or Asian British (Bangladeshi origin)  Asian or Asian British (Chinese origin)  Asian or Asian British (Other Asian origin)  Black or Black British  Mixed Race  Other | 180  150 (83%)  0  8 (4%)  1 (1%)  2 (1%)  (2 (1%)  0  1 (1%)  0  2 (1%)  8 (4%)  6 (3%) | 71  59 (41%)  0  4 (6%)  1 (1%)  1 (1%)  1 (1%)  0  1 (1%)  0  0  2 (2%)  2 (2%) |
| Number of children in household  1  2  3  4  5 | 179  35 (19%)  102 (57%)  33 (18%)  6 (3%)  2 (1%) | 70  13 (18%)  45 (63%)  9 (13%)  3 (4%)  0 |
| Child’s birth order  First born  Second born  Third born  Other | 180  93 (52%)  61 (34%)  16 (9%)  10 (5%) | 71  40 (56%)  23 (32%)  5 (7%)  3 (4%) |
| Child’s handedness  Left-handed  Right-handed | 180  16 (9%)  164 (91%) | 71  6 (9%)  65 (92%) |
| Respondent’s relationship to child  Mother  Father | 180  172 (96%)  8 (4%) | 71  68 (96%)  3 (4%) |
| Child’s Primary Caregiver?  Yes  No  Shared | 180  145 (81%)  0  35 (19%) | 71  56 (79%)  0  15 (21%) |
| Parent age  0-29  30-40  41-50  51+ | 174  15 (8%)  133 (73%)  26 (14%)  0 | 69  6 (8%)  50 (70%)  13 (18%)  0 |
| Parent marital status  Single  Married  Divorced  Other | 180  13 (7%)  142 (79%)  5 (3%)  20 (11%) | 71  2 (3%)  61 (86%)  0  8 (11%) |
| Parent employment status  Employed full-time  Employed part-time  Full-time home-maker  Unemployed  Other | 180  25 (14%)  76 (42%)  48 (27%)  6 (3%)  25 (14%) | 71  7 (10%)  34 (48%)  19 (27%)  1 (1%)  10 (14%) |
| Parent level of education  Primary School  GCSEs  A’ Levels  College Course Certificate  Bachelors Degree  Masters Degree  Postgraduate Degree | 180  2 (1%)  15 (8%)  11 (6%)  21 (12%)  84 (47%)  29 (16%)  18 (10%) | 71  1 (1%)  3 (4%)  3 (4%)  10 (14%)  38 (54%)  10 (14%)  6 (9%) |

Reasons for not completing the EEG task are shown in Table S2 below.

**Table S2.** Reasons that children did not complete EEG task.

| Parent didn’t consent | Child didn’t assent | Child didn’t tolerate net/ withdrew assent | Technical problems or time constraints | Team decided not feasible (i.e. fidgety child) | Medical concerns (i.e. allergy, scrape on face) | Other | Did EEG |
| --- | --- | --- | --- | --- | --- | --- | --- |
| 3 | 31 | 37 | 3 | 3 | 2 | 4 | 97 |

There were no significant differences between those who completed the EEG task and those who did not according to anxiety or BI (*p* > .05). After pre-processing, 71 participants (3.42 - 4.67 years (M = 4.01, SD = 0.27; 43 female)) had EEG data that were suitable for analysis. There were no significant differences according to anxiety, BI, age, or sex between those with usable EEG data and those whose EEG data were removed during pre-processing (*p* > .05), and results are consistent when BI and anxiety are compared between the full sample who did not complete the EEG task and those that did.

**Procedure Baseline**

During the lab session, the Lab-TAB was completed prior to the EEG task. For the EEG task, participants were told that they were going to play a game on the computer wearing an ‘astronaut hat’. They were given an age-appropriate video to watch whilst the cap was prepared and the EEG recording was set-up. Participants were told that faces would appear on the screen and their job was to sit as still as possible whilst looking at the faces. After each block, participants played a short ipad game with the experimenter as a reward before continuing to the next block.

**Apparatus and Materials**

**Parent report of BI.** Parents completed the Behavioural Inhibition Questionnaire (BIQ; Bishop, Spence, & McDonald, 2003) which asks how often certain behaviors occur for their child (e.g. ‘approaches new situations or activities very hesitantly’; ‘Is very quiet around new (adult) guests to our home’). The questionnaire included 30 items, scored on a 7-point Likert scale (1-7) and has good psychometric properties, adequate internal consistency, moderate stability of time and strong construct validity (Bishop et al., 2003; Kim et al., 2011). In this sample, Cronbach’s alpha = .96. The total score indicates overall level of BI, with higher scores indicating more inhibited behavior. The descriptive statistics for the total BIQ score were as follows: M = 118, SD = 31.86, range = 54 – 205.

**Observation of BI.** Two fear episodes from the Laboratory Temperament Assessment Battery (Lab-TAB) were used to observe BI. The Risk Room and Stranger Approach were selected as being most relevant to the construct of BI. The Lab-TAB is a standardized observational measure of temperament in 3-5-year-old children (Gagne, Van Hulle, Aksan, Essex, & Goldsmith, 2011). The risk room had two phases. In the first, the child was given free time to explore and play in an unfamiliar room that contained five novel/ambiguous toys (e.g. balance beam; tunnel; black box; wolf mask; steps and mattress). In the second, a research assistant entered the room and asked the child to play with each toy. A parent (typically the mother) was in the room at all times but was asked to sit in a specific seat in the corner and to remain as neutral as possible so that we could observe their child’s behavior. During the Lab-TAB stranger approach episode, the child was approached by a ‘stranger’ as they waited alone in the room. The Lab-TAB manual specifies a male stranger, but as this was not feasible, an unfamiliar female researcher acted as the stranger and wore a boiler suit with a hood to appear slightly more threatening.

Episodes were videotaped and coded according to the Lab-TAB manual. During free play phase one, the following were coded: latency to touch all objects, total number of objects touched, total time spent playing with objects, latency to first vocalization, wary fearful facial affect, tentativeness of play, and approaching parent. During phase two, compliance with requests to play, latency of compliance, tentativeness of play, referencing of parent, referencing of experimenter, distress vocalizations, wary/fearful facial affect, and a number of additional prompts required were coded. During the stranger approach episode, the following were coded across 9 epochs that aligned with specific questions and actions by the stranger (e.g. stranger asks ‘have you been here before?’ and ‘are you playing with lots of toys?’) and then averaged across epochs to give a score for each: intensity of fear expression, intensity of vocal distress, intensity of activity decrease, intensity of approach behaviors, gaze aversion, intensity of verbal hesitancy, nervous fidgeting. Further details of the procedure for these episodes can be found in the Lab-TAB manual (Goldsmith, Reilly, Lemery, Longley, & Prescott, 1999).

Scores for each coding criteria were reverse coded as necessary so that higher scores indicated more inhibited responses. Scores for every code were then converted into z-scores individually and then averaged to give a single BI score (M = -0.04, SD = 0.44, range = -0.74-1.53), in line with previous research (Gagne et al., 2011; Goldsmith et al., 1999). For information, the descriptive statistics for the risk room (RM) and stranger approach (SA) were as follows: RR: M = -0.008, SD = 0.69, range = -0.81 – 3.55; SA: M = 0.003, SD = 0.48, range = -1.38 – 1.53. To check reliability, a secondary coder coded 24% of the Lab-TAB assessments. Interrater reliability was good to excellent (ICC(2,1) = .95; 95% CI =.91 to .98). Both coders were blind to the child’s anxiety scores.

**Overall BI score.** Children’s total scores on the BIQ and their total observed BI scores were correlated (r = .32, p < .001) and were combined by converting both measures into z-scores and averaging. Of the 71 participants with useable EEG data, one was missing a score for BI due to technical problems whilst measures were being completed.

**Parent report of anxiety symptoms.** The Preschool Anxiety Scale (PAS) (4) is designed for parents of children aged 2.5-6.5 years. The scale includes 28 items (e.g. ‘Has trouble sleeping due to worries’ (Generalized Anxiety); ‘Is afraid of talking in front of the class’ (Social Anxiety)) that contribute to five subscales (OCD, Social Anxiety, Separation Anxiety, Physical Injury Fears, Generalized Anxiety) and a total score which provides an overall measure of child anxiety symptoms. Items are scored on a 5-point Likert scale from ‘Not at all True’ to ‘Very Often True’ (0-4). The measure has good construct validity, satisfactory internal consistency and good cross-informant and test-retest reliability (4). Cronbach’s alpha at both baseline and follow-up = .91. There was one participant missing a PAS score at baseline, six at the first follow-up and four at the second follow-up. The descriptive statistics for the PAS are shown in Table S3 below.

**Table S3.** Descriptive statistics for the Preschool Anxiety Scale at T1, T2 and T3

|  | Mean | SD | Range |
| --- | --- | --- | --- |
| T1 | 23.79 | 14.47 | 1 - 78 |
| T2 | 22.43 | 13.65 | 0 - 63 |
| T3 | 20.69 | 14.85 | 0 - 91 |

**EEG Pre-processing**

The EEG data were highpass filtered at 0.3 Hz and lowpass filtered at 40 Hz using EEGLAB v14.1.2 (5) FIR filters. Artifact-laden channels were identified and removed using the EEGLAB plug-in FASTER (6). Independent component analysis (ICA) was then performed on an identical copy of the dataset. Before ICA, this dataset copy was highpass filtered at 1Hz and segmented into 1s epochs. In the copy of the dataset, noisy segments were rejected using a combined voltage threshold of ±1000μV and spectral threshold (range -100dB to +30dB) within the 20-40Hz frequency band to remove EMG-like activity. After ICA decomposition, independent components were transferred from the copy ICA dataset to the original dataset. Artifactual independent components were removed from the original dataset using the EEGLAB adjusted ADJUST plugin (7) . Trials in which the child was not attending to the screen or was making large movements were also identified from videos and rejected. Missing channels were then interpolated and the data were average referenced. The data were then segmented into epochs from 200ms before stimulus onset to 2000ms after, and baseline-corrected using the pre-stimulus time period using ERPLab v7.0.0 (8). After segmenting, a moving window peak-to-peak voltage threshold of 150 µV with a window size of 200ms and a step size of 50ms was used to remove epochs contaminated by artifacts. Trials were then averaged within condition for each subject, and a 30Hz Butterworth lowpass filter (order = 2) was applied.

Time windows for analysis of each ERP component were determined empirically using the aggregate grand average from trials (AGAT), allowing data driven time window selection without inflating Type I errors (9). This involves averaging all unfiltered trials from all subjects in all conditions, rather than successive averaging within subject, within condition, and then across conditions. For each component, the window was centred on the corresponding extrema in the AGAT waveform, and extended from halfway between the previous extrema and halfway to the following extrema. This yielded time windows of 90 – 182ms for P1, 188 – 318ms for P2, and 312 – 470ms for N2. For each component, the mean amplitude was computed within the corresponding cluster and time window from the trial-averaged, filtered data for each participant.

**Statistical Analysis**

We probed significant interactions using the Johnson-Neyman technique (10). This method, unlike traditional techniques probing at arbitrary values such as plus or minus one standard deviation around the average of the predictor, provides a data-driven approach to decomposing significant interactions between two continuous variables via determination of the specific value of predictor 1 at which the relationship between predictor 2 and the dependent variable changes; i.e. determines a region-of-significance at which the simple slope becomes significant and gives confidence bands that signify the precision of the simple slope estimate (see Bauer and Curran (11); Miller et al. (12)).

**Post-hoc Power Analyses**

We calculated post-hoc power analyses for all significant main effects and interactions using the R package simr (v1.0.6; (13)), which calculates power for linear mixed models through simulation, with the model coefficients representing the simple effect sizes. Specifically, for models including angry biases: i) P1, for the main effect of BI, power to detect the effect size we observed (5.9) with our current sample size is 95.9%; for the angry bias-poly1 interaction, power to detect the effect size we observed (-5.6) with our current sample size is 58.3%; ii) N2, for the main effect of BI, power to detect the effect size we observed (5.3) with our current sample size is 92%; for the angry bias-BI interaction, power to detect the effect size we observed (4.4) with our current sample size is 57.2%. For the model including happy bias: P1, for the main effect BI, power to detect the effect size we observed (7.2) with our current sample size is 99.5%; for the happy bias-BI interaction, power to detect the effect size we observed (-12) with our current sample size is 73.5%; happy bias-poly1 interaction, power to detect the effect size we observed (-8) with our current sample size is 83.8%.

**REFERENCES**

1. Bishop G, Spence SH, McDonald C. Can parents and teachers provide a reliable and valid report of behavioral inhibition? Child development. 2003;74(6):1899-917.

2. Gagne JR, Van Hulle CA, Aksan N, Essex MJ, Goldsmith HH. Deriving childhood temperament measures from emotion-eliciting behavioral episodes: scale construction and initial validation. Psychological assessment. 2011;23(2):337.

3. Dodd HF, Rayson H, Ryan Z, Bishop C, Parsons S, Stuijfzand B. Trajectories of Anxiety When Children Start School: The Role of Behavioral Inhibition and Attention Bias to Angry and Happy Faces. Journal of abnormal psychology (1965). 2020;129(7):701-12.

4. Spence SH, Rapee R, McDonald C, Ingram M. The structure of anxiety symptoms among preschoolers. 2001.

5. Delorme A, Makeig S. EEGLAB: an open source toolbox for analysis of single-trial EEG dynamics including independent component analysis. Journal of neuroscience methods. 2004;134(1):9-21.

6. Nolan H, Whelan R, Reilly RB. FASTER: fully automated statistical thresholding for EEG artifact rejection. Journal of neuroscience methods. 2010;192(1):152-62.

7. Mognon A, Jovicich J, Bruzzone L, Buiatti M. ADJUST: An automatic EEG artifact detector based on the joint use of spatial and temporal features. Psychophysiology. 2011;48(2):229-40.

8. Lopez-Calderon J, Luck SJ. ERPLAB: an open-source toolbox for the analysis of event-related potentials. Frontiers in human neuroscience. 2014;8:213.

9. Brooks JL, Zoumpoulaki A, Bowman H. Data‐driven region‐of‐interest selection without inflating Type I error rate. Psychophysiology. 2017;54(1):100-13.

10. Johnson PO, Neyman J. Tests of certain linear hypotheses and their application to some educational problems. Statistical research memoirs. 1936.

11. Bauer DJ, Curran PJ. Probing interactions in fixed and multilevel regression: Inferential and graphical techniques. Multivariate behavioral research. 2005;40(3):373-400.

12. Miller JW, Stromeyer WR, Schwieterman MA. Extensions of the Johnson-Neyman technique to linear models with curvilinear effects: Derivations and analytical tools. Multivariate behavioral research. 2013;48(2):267-300.

13. Green P, MacLeod CJ. SIMR: an R package for power analysis of generalized linear mixed models by simulation. Methods in Ecology and Evolution. 2016;7(4):493-8.
